# Effects of Egg Consumption on Subjects with SLD or Hypertension: A MICOL Study

**DOI:** 10.3390/nu16030430

**Published:** 2024-01-31

**Authors:** Rossella Tatoli, Caterina Bonfiglio, Francesco Cuccaro, Angelo Campanella, Sergio Coletta, Pasqua Letizia Pesole, Gianluigi Giannelli, Rossella Donghia

**Affiliations:** 1 National Institute of Gastroenterology—IRCCS “Saverio de Bellis”, 70013 Castellana Grotte, Italy; rossella.tatoli@irccsdebellis.it (R.T.); catia.bonfiglio@irccsdebellis.it (C.B.); angelo.campanella@irccsdebellis.it (A.C.); sergio.coletta@irccsdebellis.it (S.C.); letizia.pesole@irccsdebellis.it (P.L.P.); gianluigi.giannelli@irccsdebellis.it (G.G.); 2 Local Health Unit—Barletta-Andria-Trani, 76121 Barletta, Italy; francescocuccaroepi@gmail.com

**Keywords:** SLD, HTN, diet, eggs

## Abstract

Background: Steatotic liver disease (SLD) is defined as a fat accumulation in more than 5% of hepatocytes; it can progress to non-alcoholic steatohepatitis (NASH), associated with an increased state of inflammation. The aim of this study was to explore the protective effects of eating eggs and any association with SLD and hypertension (HTN). Methods: The study cohort included 908 participants assessed in the fourth recall of the MICOL study, grouped into four groups, based on NALFD and/or HTN. Results: The prevalence of HTN and SLD among participants was 31.61%. Overall, the results indicated a statistical significance of egg consumption, showing a protective role against the two disease conditions, in both the raw and adjusted models (RRR = 0.34, *p* = 0.009, 0.15 to 0.76 95% C.I.). Conclusions: Many differences were found among the groups, and the protective role of eating eggs was amply demonstrated. We can conclude that it is unwise to demonize the intake of this food and its nutritional properties, in contrast with previous reports in the literature.

## 1. Introduction

Steatotic liver disease (SLD) is defined as a fat accumulation in more than 5% of hepatocytes, in the absence of significant alcohol consumption (30 g per day for men and 20 g per day for women) or other chronic liver diseases [1]. In this condition, hepatocytes present a ballooning appearance; this lesion is characteristic of SLD [1]. The overlap of some risk factors or injuries to the liver may promote the progression to non-alcoholic steatohepatitis (NASH), in which the steatosis is associated with a state of necroinflammation and fast fibrosis progression, unlike in SLD [1].

The increasing prevalence of SLD has made it a significant public health problem worldwide [2]. It is the primary cause of chronic liver disease in Europe [3] and affects 44% of inhabitants of Western countries [4].

SLD is known to be strongly linked to metabolic syndrome and can be considered the hepatic manifestation [5]. The increasing prevalence of metabolic syndrome and its related conditions, such as insulin resistance, hypertension (HTN), and dyslipidemia, predispose many people to the risk of developing SLD [6].

Together with SLD, HTN is another important health problem because it is one of the major causes of cardiovascular disease [7]. Among cardiovascular diseases, HTN is one of the main risk factors predisposing to fatal complications [8], and HTN diagnoses were estimated to increase by about 60% between 2000 and 2025 [9]. 

Diet and eating habits play a central role in the prevention and management of both SLD and HTN. They are considered modifiable risk factors for the development of HTN [10] and the only recommended treatment for SLD [11].

Dietary components can influence the pathophysiology of diseases [12,13,14,15]. Special attention should be paid to dietary cholesterol, which is a modifiable risk factor for HTN [16], while the role of this dietary component concerning the risk and progression of SLD is still controversial [17,18].

Eggs are one of the most important dietary sources of cholesterol, present in the egg yolk at 180–225 mg per egg [19]. Many societies and organizations recommend limiting egg consumption to avoid raising circulating cholesterol levels [20]. The American Heart Association recommends reducing egg intake unless other sources of dietary cholesterol are limited [21].

The high cholesterol content (an average egg provides about 70% of the recommended daily value) has led to eggs often being demonized, despite their being rich in other nutrients that can provide beneficial effects to human health. They are a good source of minerals, vitamins, proteins, and unsaturated fatty acids [22,23,24,25].

Very few studies have analyzed the relationship between egg consumption and the risk of diseases, in particular SLD, and the results are inconsistent [26,27].

The present study aims to investigate the effect of egg consumption on the risk of developing steatosis, HTN, or both conditions in an older Southern Italian population.

## 2. Materials and Methods

### 2.1. Study Design and Population

This cross-sectional study involved 980 subjects aged over 60 years. The study sample is a sub-sample from the Multicenter Italian study on Cholelithiasis (MICOL). MICOL recruitment started in 2017 among subjects listed on the electoral register of Castellana Grotte, Southern Italy. The present study is based on data from MICOL IV, a recall of MICOL III patients [28]. The methodological details of this population-based study have been described elsewhere [29,30]. All participants provided informed consent before the examination. The study was approved in line with the ethical standards of the institutional research committee of the National Institute of Gastroenterology and Research Hospital “S. de Bellis” in Castellana Grotte, Italy (DDG-CE 782/2013. The date of approval was Prot. n.144/C.E. of 15/04/2019). The IRB of the head institution, the National Institute of Gastroenterology and Research Hospital “S. de Bellis” in Castellana Grotte, Italy, approved the study. The study was conducted in accordance with the Helsinki Declaration of 1975 and adhered to the “Standards for Reporting Diagnostic Accuracy Studies” (STARD) guidelines (http://www.stard-statement.org/ Accessed on 5 October 2019). The manuscript is organized according to the “Strengthening the Reporting of Observational Studies in Epidemiology-Nutritional Epidemiology” (STROBE-nut) guidelines https://www.strobe-nut.org/ Accessed on 5 October 2019).

Our study sample was divided into four categories: No Steatosis and No HTN (a); No Steatosis and Yes HTN (b); Yes Steatosis and No HTN (c); Yes Steatosis and Yes HTN (d). Steatosis was detected with a standardized ultrasound examination [31], while HTN was defined according to specific guidelines [32].

### 2.2. Lifestyle, Clinical and Dietary Assessment

Lifestyle and anthropometric assessments were evaluated by a physician during an examination at the study center. Smoking status was assessed with the single question, “Are you a current smoker?”. The level of education was expressed as years of schooling.

Body mass index (BMI) was calculated as kg/m^2^. Height and weight measurements were performed using a Seca 220 stadiometer and a Seca 711 scale.

Blood was collected from the subjects the morning after an overnight fast. The serum was separated into aliquots. One aliquot was immediately stored at −80 °C. The second aliquot was used to test serum biochemical markers by standard laboratory techniques in our laboratory. Other aliquots were stored for use as necessary. Diet and eating habits were investigated with a validated food frequency questionnaire, administered during the visit, and each food (86 validated foods) was converted to mean daily intake in grams [33].

### 2.3. Statistical Analysis

Patient characteristics are reported as mean and standard deviation (M ± SD) for continuous variables, and as frequency and percentages (%) for categorical variables. To test the association between the independent groups (SLD (No)/HTN (No), SLD (No)/HTN (Yes), SLD (Yes)/HTN (No), and SLD (Yes)/HTN (Yes)), a Chi-square test was used for categorical variables, while the Kruskal–Wallis equality rank test was used to compare more than two independent groups. Dunn’s test and the proportion test were performed for multiple pairwise comparisons.

We estimated a multiple multinomial logistic regression model using the four-category outcome variable described above as the dependent variable and egg consumption (both continuous and categorical) determinants as predictors. The models were also adjusted for some covariates (Model 1—for age and gender; Model 2—for age, gender, and daily kcal intake), and estimated coefficients were transformed to relative-risk ratios (RRR).

To test the null hypothesis of non-association, the two-tailed probability level was set at 0.05. The analyses were conducted with StataCorp (2023. Stata Statistical Software: Release 18. College Station, TX, USA: StataCorp LLC.), while RStudio (“Prairie Trillium” Release) was used for the plots.

## 3. Results

Cohort characteristics stratified by the disease group combinations are shown in Table 1.

Males are more prevalent in the SLD and no-HTN group compared to both the healthy group (60.29% vs. 49.15%, *p* = 0.019) and the HTN-only group (60.29% vs. 50.00%, *p* = 0.04). The mean age is higher in the diseased group than in both the healthy group (69.24 ± 8.44 vs. 61.34 ± 12.16, *p* < 0.0001) and the SLD-only group (69.24 ± 8.44 vs. 61.72 ± 10.35, *p* < 0.0001). The same applies for the HTN-only group compared to the healthy group (70.54 ± 9.33 vs. 61.34 ± 12.16, *p* < 0.0001), while there was a statistically significant number of younger subjects in the NALFD-only group compared to the HTN-only group (61.72 ± 10.35 vs. 70.54 ± 9.33, *p* < 0.0001).

The level of education was statistically significantly associated with the disease conditions in all multiple comparisons with the exception of the group with both disease conditions versus HTN-only subjects. BMI was lower in healthy subjects and was statistically higher values in the groups with the disease conditions, and the highest value was found in the group with both (31.56 ± 5.39). The same trend was also observed for diabetes and metabolic syndrome. Diabetes was more prevalent in the group of patients with both conditions, showing a prevalence of 22.65%, except in comparison with subjects with SLD and with only HTN (8.13% vs. 13.64%, *p* = 0.08). Subjects with metabolic syndrome were less prevalent in the group with neither disease condition, to a statistically significant degree (*p* < 0.005), with the exception of the NALFD vs. HTN groups (40.19% vs. 43.75, *p* = 0.48). As shown in Supplementary Table S1, blood test results showed higher glucose concentrations in the group with both conditions than in the other groups, while HDL tended toward a statistically significant decrease (*p* < 0.0001).

Hematocrit (HCT), mean corpuscular volume (MCV), white blood cells (WBCs), neutrophils, hemoglobin A1c (HbA1c), gamma-glutamyl transferase (GGT), and C-reactive protein (CRP) tended toward a statistically significant increase in the last group, both in general and in multiple comparisons.

The other parameters showed no defined trend, even if the variations were significant, and even in multiple comparisons.

**Figure 1:**
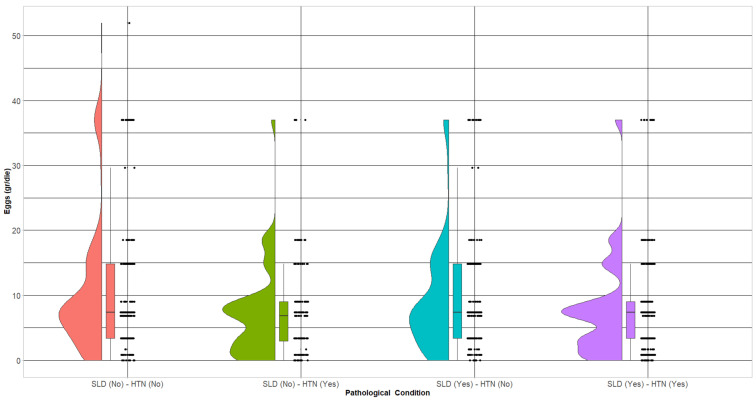
Half-violin plot of daily egg consumption by group.

Figure 1 and Table 2 illustrate egg consumption both as grams/day and as the number of eggs per week.

There was a significant difference between the groups in terms of daily consumption (*p* = 0.0001) which was also reflected in multiple comparisons, where the greatest consumption was in healthy subjects without either disease, followed by those affected by SLD; weekly intake was also statistically significantly different (*p* = 0.004). 

The relative nutrients, fatty acids, and amino acid intake is reported in Supplementary Table S2.

The multinomial logistic regression models are reported in Table 3.

Egg intake (g/die) showed a protective role in subjects with only HTN (RRR = 0.95, *p* < 0001, 0.93 to 0.98 C.I. 95%) and with SLD (RRR = 0.96, *p* = 0.001, 0.94 to 0.98 C.I. 95%), and a consumption > 3 (week) was more protective for these groups (RRR = 0.21, *p* = 0.005, 0.07 to 0.62 C.I. 95%, and RRR = 0.34, *p* = 0.006, 0.15 to 0.73 C.I. 95%, respectively) in the unadjusted model. The positive association was confirmed in the models adjusted for covariates.

## 4. Discussion

The present study shows a positive effect of egg consumption in an older population living in the Southern Mediterranean area. Our analyses revealed a protective power of eggs, which increases with dietary consumption. This relationship was also significant after adjustment for age, gender, and daily kcal intake.

In the scientific literature, data on the link between eggs consumption and SLD are few, inconsistent, and controversial.

Some previous studies associated egg consumption with an increased risk of NALFD [30]. In these, the positive link between egg consumption and SLD was explained by the high cholesterol content of eggs [34,35].

Some studies have shown that a higher cholesterol consumption is associated with SLD and its exacerbation to NASH [35,36]. Baumgartner et al. [26] has shown that daily egg consumption increases serum cholesterol and LDL-C concentrations in women [37] On the contrary, some randomized controlled trials observed that egg intake did not substantially change plasma TC, low-density lipoprotein cholesterol (LDL-C), HDL-C levels [38,39] or the TC/HDL-C ratio [39,40].

Therefore, reducing egg consumption as a single food may not be so important for reducing the risk of disease. In our opinion, it is more important to frame egg consumption within the individual’s eating habits. Egg preparation methods and concurrent consumption of other foods (e.g., bacon vs. vegetables) may partly account for the differences. Dietary fiber is known to reduce the intestinal absorption of cholesterol [41,42]. For this reason, consuming a dish with mixed vegetables and eggs has a different effect on serum cholesterol concentrations than a low-fiber dish such as eggs and bacon. Mokhtari et al. [26] used this argument to explain how consumption of more than four eggs per week was not significantly associated with the risk of SLD [43]. Our study population is linked to food traditions within the Mediterranean dietary pattern. They have healthy eating habits, and vegetables are a constant in their diet. The cooking methods used are simple, healthy, and without added fat [40]. For example, the preparation of vegetable timbale, a dish made of eggs and vegetables (zucchini, mushrooms, spinach, etc.), baked in the oven or in a non-stick pan without adding fat, is extremely frequent in our study population’s diet. Genetic factors also appear to affect cholesterol absorption [44]. Homeostatic control of cholesterol absorption adapts to dietary cholesterol intake, decreasing the amount of cholesterol absorbed at higher dietary intake levels and downregulating endogenous cholesterol synthesis [45,46]. Cholesterol aside, eggs are nutritionally an excellent source of beneficial nutrients. Eggs provide high biological value protein, monounsaturated and polyunsaturated fatty acids, arginine—a precursor to nitric oxide, carotenoids, minerals, and vitamins [47,48].

A current topic of interest is the effect of methyl donor food deficiency, including VIT B12 and folic acid, on the development of SLD [49]. A recent randomized, double-blind controlled trial evaluated the effects of vitamin B12 supplementation on serum liver enzymes, the degree of hepatic steatosis, and metabolic profiles in patients with SLD [50]. A reduction in the steatosis score and liver enzymes, especially ALT, was observed in the VIT B12-treated group. In another study, patients with liver steatosis had lower VIT B12 levels than healthy subjects [51]. A study conducted in rats suggested that the supplementation of folates and VIT B12 could improve obesogenic diet-induced hepatic triglyceride accumulation [52].

Chicken eggs have a non-negligible content of folates and VIT B12 [www.bda-ieo.it. (accessed on 9 November 2023)]. This could explain the protective effect of regular, moderate consumption.

A protective role of eggs has also been found in HTN [53]. Previous studies considered peptides with antihypertensive properties that can stimulate the release of nitric oxide and prostacyclins and inhibit angiotensin-converting enzyme (ACE) activity and endothelium-dependent vasodilatory activity [53]. However, despite the number of studies already published, more well-designed prospective cohort studies are needed to further examine and support this finding [53].

## 5. Conclusions

In conclusion, the results from our study show a protective role of eggs both against HTN only and associated with SLD. In the study population, the risk of developing these pathological conditions decreased linearly with increased egg consumption (consumption of >3 eggs per week).

In our view, it is unwise to demonize a nutritionally noble food like eggs. It is necessary to inform people about the wise consumption of eggs in order to take full advantage of their nutritional properties. However, further studies are needed to support our results and also to define egg consumption in populations other than those living in Mediterranean areas.

### Strengths and Limitations

Among the strengths of the present study are the ample cohort and the generalizability of the results to populations of the Southern Mediterranean. To the best of our knowledge, the scientific literature lacks data and consistent results on the link between egg consumption and SLD. There are currently few studies evaluating this topic, and none were conducted on an older Mediterranean population.

Another important strength is the FFQ, which is the dietary assessment method most commonly used to study diet and eating habits. The FFQ is very complete and detailed, allowing us to obtain all the necessary information about the eating habits of our study population.

However, there are also some limitations. The main one is the nature of the study, which, being cross-sectional, does not clarify the directionality of an association. In addition, the present study only considered the effect of whole-egg consumption; it would be interesting to study the effect of the separate consumption of egg whites and yolks.

## Figures and Tables

**Table 1:** Epidemiological and clinical characteristics of patients with SLD or HTN (*n* = 908).

Parameters *	Disease Condition				*p* ^^^	Multiple Comparisons ^ψ^					
	SLD (No)/ HTN (No) (n = 236) (a)	SLD (No)/ HTN (Yes) (n = 176) (b)	SLD (Yes)/ HTN (No) (n = 209) (c)	SLD (Yes)/ HTN (Yes) (n = 287) (d)		(b) vs. (a)	(c) vs. (a)	(d) vs. (a)	(c) vs. (b)	(d) vs. (b)	(d) vs. (c)
Gender (M) (%)	116 (49.15)	88 (50.00)	126 (60.29)	164 (57.14)	0.05 ^¥^	0.86 ^†^	0.01 ^†^	0.48 ^†^	0.04 ^†^	0.13 ^†^	0.48 ^†^
Age (yrs)	61.34 ± 12.16	70.54 ± 9.33	61.72 ± 10.35	69.24 ± 8.44	0.001	<0.0001	0.44	<0.0001	<0.0001	0.07	<0.0001
Degree of Education (%)					<0.001 ^¥^						
No	59 (25.11)	77 (43.75)	47 (22.71)	114 (40.00)		0.0001 ^†^	<0.0001 ^†^	0.0001 ^†^	0.04 ^†^	0.13 ^†^	0.48 ^†^
Elementary School	68 (28.94)	43 (24.43)	81 (39.13)	89 (31.23)		0.31 ^†^	0.002 ^†^	0.07 ^†^	0.002 ^†^	0.12 ^†^	0.07 ^†^
Secondary School	75 (31.91)	29 (16.48)	52 (25.12)	45 (15.79)		0.0004 ^†^	0.04 ^†^	0.01 ^†^	0.04 ^†^	0.84 ^†^	0.01 ^†^
High School	27 (11.49)	6 (3.41)	17 (8.21)	10 (3.51)		0.003 ^†^	0.05 ^†^	0.02 ^†^	0.05 ^†^	0.95 ^†^	0.02 ^†^
Degree	6 (2.55)	21 (11.93)	10 (4.83)	27 (9.47)		0.0001 ^†^	0.01 ^†^	0.05 ^†^	0.01 ^†^	0.40 ^†^	0.05 ^†^
Smoker (Yes) (%)	36 (15.32)	18 (10.29)	33 (15.79)	31 (10.84)	0.18 ^¥^	0.14 ^†^	0.89 ^†^	0.13 ^†^	0.11 ^†^	0.85 ^†^	0.10 ^†^
BMI (kg/m^2^)	25.27 ± 3.32	27.32 ± 4.20	28.80 ± 4.33	31.56 ± 5.39	0.0001	<0.0001	<0.0001	<0.0001	0.0001	<0.0001	<0.0001
Diabetes (Yes) (%)	10 (4.26)	24 (13.64)	17 (8.13)	65 (22.65)	<0.001 ^¥^	0.0006 ^†^	0.09 ^†^	<0.0001 ^†^	0.08 ^†^	0.001 ^†^	<0.0001 ^†^
MetS (Yes) (%)	37 (15.68)	77 (43.75)	84 (40.19)	198 (68.99)	<0.001 ^¥^	<0.0001 ^†^	<0.0001 ^†^	<0.0001 ^†^	0.48 ^†^	<0.0001 ^†^	<0.0001 ^†^

* As mean and standard deviation for continuous variables, and as frequency and percentage (%) for categorical variables. ^^^ Kruskal–Wallis equality of populations rank test; ^¥^ Chi-square test; ^ψ^ Dunn’s test of multiple comparisons; ^†^ Proportion test. Abbreviations: SLD, Steatotic Liver Disease; HTN, Hypertension; BMI, Body Mass Index; MetS, Metabolic Syndrome.

**Table 2:** Eggs consumption stratified by SLD or HTN.

Parameters *	Disease Condition				*p* ^^^	Multiple Comparisons ^ψ^					
	SLD (No)/ HTN (No) (n = 236) (a)	SLD (No)/ HTN (Yes) (n = 176) (b)	SLD (Yes)/ HTN (No) (n = 209) (c)	SLD (Yes)/ HTN (Yes) (n = 287) (d)		(b) vs. (a)	(c) vs. (a)	(d) vs. (a)	(c) vs. (b)	(d) vs. (b)	(d) vs. (c)
Eggs (g/die)	10.43 ± 9.85	7.23 ± 6.98	9.45 ± 8.77	7.82 ± 7.63	0.001	0.0005	0.12	0.0007	0.02	0.30	0.03
Eggs (n#/week) (%)					0.004 ^¥^						
<2	168 (71.19)	146 (82.95)	147 (70.33)	227 (79.09)		0.005 ^†^	0.84 ^†^	0.04 ^†^	0.004 ^†^	0.31 ^†^	0.02 ^†^
2–3	46 (19.49)	26 (14.77)	48 (22.97)	50 (17.42)		0.21 ^†^	0.37 ^†^	0.55 ^†^	0.04 ^†^	0.45 ^†^	0.12 ^†^
>3	22 (9.32)	4 (2.27)	14 (6.70)	10 (3.48)		0.004 ^†^	0.31 ^†^	0.005 ^†^	0.07 ^†^	0.64 ^†^	0.10 ^†^

* As Mean and Standard Deviation for continuous variables, and as frequency and percentage (%) for categorical variables. ^^^ Kruskal–Wallis equality of populations rank test; ^¥^ Fisher’s exact test; ^ψ^ Dunn’s test of multiple comparisons; ^†^ Proportions test.

**Table 3:** Multinomial logistic regression analysis of disease condition categories using egg daily intake or eggs per week.

Parameters	SLD (No) and HTN (Yes) ^¥^				SLD (Yes) and HTN (No) ^¥^				SLD (Yes) and HTN (Yes) ^¥^			
	RRR	se (RRR)	*p*	95% C.I.	RRR	se (RRR)	*p*	95% C.I.	RRR	se (RRR)	*p*	95% C.I.
Model 1												
Eggs (g/die)	0.95	0.01	<0.001	0.93 to 0.98	0.99	0.01	0.29	0.97 to 1.01	0.96	0.01	0.001	0.94 to 0.98
Eggs (n#/week)												
<2 [Ref.]	--	--	--	--	--	--	--	--	--	--	--	--
2–3	0.65	0.17	0.11	0.38 to 1.10	1.19	0.28	0.45	0.75 to 1.89	0.80	0.18	0.34	0.51 to 1.26
>3	0.21	0.12	0.005	0.07 to 0.62	0.73	0.26	0.38	0.36 to 1.47	0.34	0.13	0.006	0.15 to 0.73
Model 2												
Eggs (g/die)	0.96	0.01	0.006	0.94 to 0.99	0.99	0.01	0.33	0.97 to 1.01	0.97	0.01	0.01	0.95 to 0.99
Eggs (n#/week)												
<2 [Ref.]	--	--	--	--	--	--	--	--	--	--	--	--
2–3	0.93	0.26	0.79	0.53 to 1.62	1.22	0.29	0.40	0.76 to 1.96	1.10	0.26	0.70	0.68 to 1.76
>3	0.27	0.15	0.02	0.09 to 0.81	0.71	0.26	0.36	0.35 to 1.46	0.40	0.16	0.03	0.18 to 0.90
Model 3												
Eggs (g/die)	0.96	0.01	0.009	0.94 to 0.99	0.99	0.01	0.22	0.97 to 1.01	0.97	0.01	0.008	0.95 to 0.99
Eggs (n#/week)												
<2 [Ref.]	--	--	--	--	--	--	--	--	--	--	--	--
2–3	0.99	0.29	0.96	0.56 to 1.75	1.17	0.28	0.51	0.73 to 1.89	1.03	0.25	0.91	0.63 to 1.67
>3	0.27	0.16	0.02	0.09 to 0.84	0.62	0.23	0.19	0.30 to 1.28	0.34	0.14	0.009	0.15 to 0.76

Abbreviations: SLD, Steatotic Liver Disease (SLD); HTN, Hypertension; RRR, Relative-Risk Ratios; se (RRR), standard error of RRR; 95% C.I., Confidential Interval at 95%. ^¥^ Reference category: SLD (No) and HTN (No). Model 1, Row Model; Model 2, Adjusted for age, and gender; Model 3, adjusted for age, gender, and kcal daily intake.

## Data Availability

The original contributions presented in the study are included in the article. Further inquiries can be directed to the corresponding author.

## Supplementary Materials

The following supporting information can be downloaded at: https://www.mdpi.com/article/10.3390/nu16030430/s1, Table S1: Blood characteristics of patients with SLD or HTN; Table S2: Nutrients (mg/die) eggs intake by disease condition.
