# Supplementary material for: Effects of Egg Consumption on Subjects with SLD or Hypertension: A MICOL Study
Source: Nutrients. 2024 Jan 31;16(3):430. doi: 10.3390/nu16030430 (PMC10856908; doi:10.3390/nu16030430)
Supplement: Supplementary file 1 [file nutrients-16-00430-s001.zip › nutrients-2824824-supplementary.pdf]

Table S1. Blood characteristics of patients with SLD or HTN.

Parameters *	SLD (No) / HTN (No) (*n*=236) *(a)*	SLD (No) / HTN (Yes) (*n*=176) *(b)*	SLD (Yes) / HTN (No) (*n*=209) *(c)*	SLD (Yes)/ HTN (Yes) (*n*=287) *(d)*	p ^	Multiple Comparisons ψ *(b) vs (a)*	*(c) vs (a)*	*(d) vs (a)*	*(c) vs (b)*	*(d) vs (b)*	*(d) vs (c)*
Glucose (mg/dL)	92.60±17.16	99.00±18.10	100.62±18.72	112.74±40.26	0.0001	<0.0001	<0.0001	<0.0001	0.15	<0.0001	0.0001
Cholesterol (mg/mL)	195.23±35.78	184.66±37.29	198.99±37.76	185.10±38.26	0.0001	0.001	0.27	0.001	0.0002	0.36	0.0002
HDL (mg/dL)	54.22±14.16	51.10±13.14	46.89±11.65	45.89±11.26	0.0001	0.009	<0.0001	<0.0001	0.0005	<0.0001	0.20
LDL (mg/dL)	126.26±30.78	115.69±31.61	129.31±33.05	115.15±33.36	0.0001	0.002	0.23	0.0003	0.0003	0.47	<0.0001
Triglycerides (mg/dL)	81.80±50.29	91.47±47.69	116.68±61.20	131.16±71.08	0.0001	0.01	<0.0001	<0.0001	<0.0001	<0.0001	0.01
Insulin (U/L)	6.39±13.13	11.59±62.62	9.48±9.12	11.23±8.19	0.0001	0.0007	<0.0001	<0.0001	<0.0001	<0.0001	0.0006
HOMA-IR	1.58±4.60	3.27±20.00	2.49±3.16	3.25±3.04	0.0001	0.0001	<0.0001	<0.0001	<0.0001	<0.0001	<0.0001
RBC (M/mcL)	4.86±0.50	4.76±0.59	4.97±0.52	4.83±0.50	0.0003	0.08	0.001	0.43	<0.0001	0.10	0.0005
Hemoglobin (g/dL)	14.03±1.49	13.59±1.68	14.32±1.53	13.96±1.48	0.0001	0.005	0.007	0.32	<0.0001	0.01	0.001
HCT (%)	41.72±3.61	42.79±3.37	43.10±3.39	43.13±3.74	0.002	0.04	0.0003	0.003	0.28	0.38	0.36
MCV (fL)	85.37±7.74	84.81±8.46	84.95±5.85	86.50±5.23	0.04	0.10	0.01	0.31	0.42	0.06	0.007
MCH (pg)	28.82±2.79	28.63±3.30	29.04±2.31	29.44±2.02	0.40	0.31	0.31	0.08	0.21	0.07	0.17
MCHC (g/dL)	33.75±1.08	33.71±1.20	34.17±1.12	34.03±1.04	0.005	0.37	0.0003	0.02	0.03	0.12	0.17
RDW-CV (%)	13.67±1.20	13.77±1.61	13.54±0.95	13.71±1.06	0.63	0.45	0.25	0.23	0.37	0.26	0.09
Platelets (K/mcL)	229.64±58.47	220.40±59.56	228.95±52.08	224.68±62.65	0.11	0.02	0.49	0.08	0.02	0.22	0.08
WBC (K/mcL)	5.77±2.78	5.81±1.58	5.88±1.32	6.35±2.20	0.0001	0.14	0.006	<0.0001	0.10	0.0002	0.009
Neutrophils (%)	57.17±8.92	58.74±8.50	56.32±8.57	58.71±8.06	0.26	0.27	0.10	0.27	0.07	0.43	0.04
Lymphocytes (%)	32.44±8.93	30.87±7.82	33.21±8.47	30.72±7.17	0.28	0.30	0.11	0.25	0.09	0.48	0.04

(continue)

Parameters *	Disease Condition				p ^	Multiple Comparisons ψ					
	SLD (No) / HTN (No) (*n*=236) (*a*)	SLD (No) / HTN (Yes) (*n*=176) (*b*)	SLD (Yes) / HTN (No) (*n*=209) (*c*)	SLD (Yes)/ HTN (Yes) (*n*=287) (*d*)		*(b) vs (a)*	*(c) vs (a)*	*(d) vs (a)*	*(c) vs (b)*	*(d) vs (b)*	*(d) vs (c)*
Eosinophils (%)	2.71±1.55	2.87±1.67	2.73±1.64	2.54±1.17	0.81	0.23	0.38	0.37	0.18	0.19	0.48
Monocytes (%)	7.10±1.56	6.89±1.54	7.25±1.61	7.51±2.03	0.36	0.23	0.21	0.10	0.10	0.06	0.31
Basophils (%)	0.58±0.34	0.62±0.36	0.49±0.24	0.51±0.28	0.08	0.33	0.01	0.11	0.03	0.10	0.23
Neutrophils (10^3/μL)	3.24±1.04	3.35±1.15	3.40±0.92	3.93±2.57	0.01	0.28	0.05	0.0004	0.29	0.04	0.04
Lymphocytes (10^3/μL)	1.98±2.93	1.72±0.58	1.99±0.66	1.90±0.50	0.002	0.36	0.0005	0.007	0.005	0.02	0.30
Monocytes (10^3/μL)	0.39±0.12	0.38±0.13	0.43±0.13	0.48±0.17	0.0001	0.31	0.002	<0.0001	0.009	0.0006	0.09
Eosinophils (10^3/μL)	0.15±0.09	0.16±0.11	0.16±0.11	0.15±0.07	0.49	0.20	0.17	0.07	0.42	0.40	0.27
Basophils (10^3/μL)	0.03±0.02	0.03±0.02	0.03±0.02	0.03±0.02	0.66	0.36	0.20	0.34	0.18	0.47	0.13
HbA1c (%)	35.43±7.15	36.49±7.43	38.21±6.20	40.09±8.70	0.0001	0.26	0.0003	<0.0001	0.05	0.007	0.11
Fractional Total Bilirubinemia (mg/dL)	0.75±0.47	0.65±0.26	0.67±0.32	0.70±0.31	0.41	0.14	0.06	0.28	0.44	0.28	0.19
Direct fractional bilirubinemia (mg/dL)	0.16±0.05	0.15±0.05	0.15±0.04	0.16±0.05	0.73	0.21	0.15	0.27	0.49	0.38	0.36
Indirect fractional bilirubinemia (mg/dL)	0.49±0.27	0.43±0.25	0.48±0.27	0.51±0.28	0.82	0.27	0.43	0.40	0.37	0.17	0.35

GOT (U/L)	22.27±12.91	22.13±9.39	21.21±6.11	23.39±10.34	0.08	0.37	0.43	0.01	0.44	0.04	0.02
SGPT (U/L)	20.88±10.96	20.66±11.42	22.49±9.09	25.90±15.44	0.0001	0.45	0.0005	<0.0001	0.0008	<0.0001	0.004
GGT (U/I)	17.00±12.22	20.52±18.85	20.52±17.31	22.99±22.35	0.0001	0.06	0.0003	<0.0001	0.05	0.001	0.09

(continue)

Parameters *	Disease Condition				p ^	Multiple Comparisons ψ					
	SLD (No) / HTN (No) (*n*=236) *(a)*	SLD (No) / HTN (Yes) (*n*=176) *(b)*	SLD (Yes) / HTN (No) (*n*=209) *(c)*	SLD (Yes)/ HTN (Yes) (*n*=287) *(d)*		*(b) vs (a)*	*(c) vs (a)*	*(d) vs (a)*	*(c) vs (b)*	*(d) vs (b)*	*(d) vs (c)*
Albumin (%)	4.14±0.25	4.11±0.24	4.16±0.25	4.11±0.24	0.47	0.14	0.42	0.15	0.12	0.38	0.12
Iron (mg/dL)	90.88±31.16	86.84±31.59	89.41±31.16	90.80±30.65	0.97	0.36	0.36	0.49	0.46	0.38	0.39
Urea (mg/dL)	37.89±9.70	43.52±14.25	37.75±9.34	42.67±12.21	0.0001	<0.0001	0.47	<0.0001	<0.0001	0.32	<0.0001
Creatinine (mg/dL)	0.76±0.22	1.01±0.89	0.79±0.15	0.85±0.21	0.0001	<0.0001	0.01	0.0001	0.01	0.14	0.07
eGFR (mL/min)	86.53±8.37	83.05±15.17	85.56±8.18	82.32±11.76	0.06	0.08	0.21	0.005	0.20	0.31	0.03
AAT (mg/dL)	192.44±37.34	186.32±37.79	194.32±43.44	191.19±36.91	0.71	0.16	0.42	0.37	0.13	0.24	0.30
Folate (ng/mL)	8.40±4.61	9.44±5.48	8.20±5.28	9.31±5.90	0.01	0.04	0.12	0.08	0.002	0.29	0.005
Vitamin B12 (pg/ml)	370.82±596.64	412.47±690.87	321.72±170.85	388.41±674.07	0.35	0.04	0.39	0.35	0.08	0.08	0.47
TSH (mUI/mL)	1139.08±1715.89	358.23±860.36	979.46±1178.61	484.11±1124.00	0.0001	<0.0001	0.47	<0.0001	<0.0001	0.26	<0.0001
FT3 (pg/mL)	3.29±0.47	3.17±0.41	3.33±0.51	3.25±0.51	0.001	0.003	0.09	0.18	<0.0001	0.02	0.01
FT4 (ng/mL)	0.86±0.16	0.88±0.15	0.91±0.64	0.88±0.19	0.24	0.24	0.25	0.10	0.09	0.34	0.03
CRP (mg/L)	0.16±0.16	0.17±0.24	0.27±0.33	0.39±0.84	0.001	0.39	0.0001	<0.0001	0.01	0.0004	0.04

* As Mean and Standard Deviation for continuous variables, and as frequency and percentage (%) for categorical. ^ Kruskal-Wallis equality of populations rank test; ¥ Chi-Square test; ψ Dunn's test of multiple comparisons; † Proportion test. Abbreviations: SDL, Steatotic Liver Disease; HTN, Hypertension; HDL, High-Density Lipoprotein; LDL, Low-Density Lipoprotein; HOMA-IR, Homeostasis Model Assessment-Estimated Insulin Resistance; RBC, Red Blood Cell; HCT, Hematocrit (he-MAT-uh-krit); MCV, Mean Corpuscular Volume; MCH, Mean Corpuscular Hemoglobin; MCHC, Mean Corpuscular Hemoglobin Concentration; RDW-CV, Red Cell Distribution Width; WBC, White Blood Cells; HbA1c, Hemoglobin A1c; GOT, Aspartate Aminotransferase; SGPT, Serum Glutamic Pyruvic Transaminase; GGT, Gamma-Glutamyl Transferase; eGFR, Estimated Glomerular Filtration Rate; AAT, Alpha-1-Antitrypsin; TSH, Thyroid Stimulating Hormone; FT3, Free Triiodothyronine; FT4, Thyroxine; PCR, CRP, C-Reactive Protein.

nutrients MDPI

Table S2. Nutrients (mg/die) eggs intake by disease condition.

Nutrients *	Disease Condition			
	SLD (No) / HTN (No) (*n*=236)	SLD (No) / HTN (Yes) (*n*=176)	SLD (Yes) / HTN (No) (*n*=209)	SLD (Yes) / HTN (Yes) (*n*=287)
Nutrients				
Iron	0.16±0.15	0.11±0.10	0.14±0.13	0.12±0.11
Calcium	5.00±4.73	3.47±3.35	4.55±4.21	3.75±3.66
Sodium	14.29±13.49	9.91±9.56	12.98±12.01	10.71±10.46
Potassium	13.87±13.10	9.62±9.28	12.60±11.66	10.39±10.15
Phosphorus	21.90±20.68	15.18±14.65	19.90±18.41	16.41±16.03
Zinc	0.12±0.12	0.09±0.08	0.11±0.10	0.09±0.09
Magnesium	1.35±1.28	0.94±0.91	1.23±1.14	1.02±0.99
Copper	0.01±0.006	0.004±0.004	0.006±0.005	0.005±0.004
Selenium	0.60±0.57	0.42±0.40	0.55±0.51	0.45±0.44
Chlorine	16.68±15.76	11.57±11.16	15.16±14.03	12.50±12.21
Iodine	5.53±5.22	3.83±3.70	5.02±4.65	4.14±4.04
Sulfur	18.77±17.73	13.01±12.56	17.06±15.78	14.07±13.74
Vitamin B1	0.09±0.009	0.006±0.006	0.008±0.008	0.007±0.007
Vitamin B2	0.03±0.03	0.02±0.02	0.03±0.03	0.02±0.02
Niacin	0.01±0.01	0.007±0.007	0.009±0.009	0.008±0.007
Vitamin B6	0.01±0.01	0.009±0.008	0.01±0.01	0.009±0.009

(continue)

Nutrients *	Disease Condition			
	SLD (No) / HTN (No) (*n*=236)	SLD (No) / HTN (Yes) (*n*=176)	SLD (Yes) / HTN (No) (*n*=209)	SLD (Yes) / HTN (Yes) (*n*=287)
Total Folates	5.21±4.92	3.61±3.49	4.74±4.38	3.91±3.82
Pantothenic Acid	0.18±0.17	0.13±0.12	0.17±0.15	0.14±0.13
Biotin	2.08±1.97	1.45±1.39	1.89±1.75	1.56±1.53
Vitamin B12	0.26±0.25	0.18±0.17	0.24±0.22	0.19±0.19
Vitamin A	22.00±20.78	15.26±14.72	20.00±18.50	16.49±16.10
Vitamin E	0.11±0.11	0.08±0.08	0.10±0.10	0.09±0.08
Vitamin D	0.18±0.17	0.13±0.12	0.16±0.15	0.14±0.13
Fatty acids				
Total Saturated Fatty Acids	0.33±0.31	0.23±0.22	0.30±0.28	0.25±0.24
Myristic Acid	0.003±0.003	0.002±0.002	0.003±0.003	0.002±0.002
Palmitic Acid	0.20±0.19	0.14±0.13	0.18±0.17	0.15±0.14
Stearic Acid	0.13±0.12	0.09±0.08	0.11±0.11	0.09±0.09
Total Monounsaturated Fatty Acids	0.27±0.25	0.19±0.18	0.24±0.23	0.20±0.20
Palmitoleic Acid	0.02±0.02	0.02±0.02	0.02±0.02	0.02±0.02
Oleic Acid	0.24±0.23	0.17±0.16	0.22±0.21	0.18±0.18
Total Polyunsaturated Fatty Acids	0.13±0.12	0.09±0.09	0.12±0.11	0.10±0.10
Linoleic Acid	0.11±0.10	0.08±0.07	0.10±0.09	0.08±0.08
Linolenic Acid	0.004±0.004	0.003±0.003	0.004±0.03	0.003±0.003
Arachidonic Acid	0.02±0.01	0.01±0.01	0.01±0.01	0.01±0.01

(continue)

Nutrients *	Disease Condition			
	SLD (No) / HTN (No) (*n*=236)	SLD (No) / HTN (Yes) (*n*=176)	SLD (Yes) / HTN (No) (*n*=209)	SLD (Yes) / HTN (Yes) (*n*=287)
Aminoacids				
Tryptophan	20.54±19.40	14.24±13.74	18.67±17.27	15.40±15.03
Threonine	64.97±61.37	45.05±43.46	59.05±54.61	48.69±47.55
Isoleucine	68.51±64.72	47.50±45.83	62.27±57.60	51.35±50.14
Leucine	108.56±102.54	75.27±72.62	98.66±91.26	81.37±79.45
Lysine	91.77±86.68	63.63±61.39	83.40±77.15	68.78±67.16
Methionine	45.57±43.05	31.60±30.49	41.42±38.31	34.16±33.35
Cystine	33.68±31.82	23.35±22.53	30.61±28.32	25.25±24.65
Phenylalanine	68.04±65.21	47.87±46.18	62.74±58.03	51.74±50.52
Tyrosine	52.45±49.55	36.37±35.09	47.67±44.09	39.31±38.39
Valina	85.82±81.07	59.51±57.41	78.00±72.15	64.33±62.81
Arginine	79.98±75.55	55.46±53.51	72.69±67.24	59.95±58.54
Histidine	31.08±29.35	21.55±20.79	28.24±26.12	23.29±22.74
Alanine	74.98±70.82	51.99±50.16	68.14±63.03	56.20±54.88
Aspartic Acid	126.08±119.09	87.42±84.34	114.59±105.99	94.50±92.27
Glutamic Acid	157.26±148.54	109.04±105.20	142.92±132.20	117.87±115.09
Glycine	43.38±40.98	30.08±29.02	39.43±36.47	32.51±31.75
Proline	51.72±48.86	35.86±34.60	47.01±43.48	38.77±37.86
Serine	87.81±82.94	60.88±58.74	79.80±73.81	65.81±64.26

* As Mean and Standard Deviation (M±SD).
